# A Bioinformatics Tool for Predicting Future COVID-19 Waves Based on a Retrospective Analysis of the Second Wave in India: Model Development Study

**DOI:** 10.2196/36860

**Published:** 2022-09-22

**Authors:** Ashutosh Kumar, Adil Asghar, Prakhar Dwivedi, Gopichand Kumar, Ravi K Narayan, Rakesh K Jha, Rakesh Parashar, Chetan Sahni, Sada N Pandey

**Affiliations:** 1 Department of Anatomy All India Institute of Medical Sciences - Patna Patna India; 2 Department of Anatomy Dr B C Roy Multispeciality Medical Research Center Indian Institute of Technology-Kharagpur Kharagpur India; 3 India Health Lead Oxford Policy Management Limited Oxford United Kingdom; 4 Department of Anatomy Institute of Medical Sciences Banaras Hindu University Varanasi India; 5 Department of Zoology Banaras Hindu University Varanasi India

**Keywords:** COVID-19, epidemiology, genomic surveillance, second wave, SARS-CoV-2

## Abstract

**Background:**

Since the start of the COVID-19 pandemic, health policymakers globally have been attempting to predict an impending wave of COVID-19. India experienced a devastating second wave of COVID-19 in the late first week of May 2021. We retrospectively analyzed the viral genomic sequences and epidemiological data reflecting the emergence and spread of the second wave of COVID-19 in India to construct a prediction model.

**Objective:**

We aimed to develop a bioinformatics tool that can predict an impending COVID-19 wave.

**Methods:**

We analyzed the time series distribution of genomic sequence data for SARS-CoV-2 and correlated it with epidemiological data for new cases and deaths for the corresponding period of the second wave. In addition, we analyzed the phylodynamics of circulating SARS-CoV-2 variants in the Indian population during the study period.

**Results:**

Our prediction analysis showed that the first signs of the arrival of the second wave could be seen by the end of January 2021, about 2 months before its peak in May 2021. By the end of March 2021, it was distinct. B.1.617 lineage variants powered the wave, most notably B.1.617.2 (Delta variant).

**Conclusions:**

Based on the observations of this study, we propose that genomic surveillance of SARS-CoV-2 variants, complemented with epidemiological data, can be a promising tool to predict impending COVID-19 waves.

## Introduction

The year 2019 had a SARS-CoV-2–driven wave of COVID worldwide that soon turned into a pandemic, and to date, this disease has killed about 65 million people [1]. Since the pandemic’s start, much policy talk has been about whether an impending COVID wave can be predicted [2]. Unfortunately, successful prediction of COVID waves has not yet been achieved. A prediction tool that can inform about an upcoming COVID wave well before time and reasonably accurately could minimize the enormous loss of life and other collateral damages.

Multiple waves at a global scale driven by SARS-CoV-2 variants, primarily Alpha, Delta [3], and, most recently, Omicron [4], have followed since the first wave. The successive SARS-CoV-2 variants showed increased transmissibility and virulence compared with the wild-type strain [3]; however, the latest Omicron variant has shown higher transmissibility and immune escape but lesser lethality compared with the Delta variant [4]. The Delta variant–driven wave was characterized by high speed of rising cases, increased oxygen demand, vaccine breakthrough [5], a highly increased proportion of severe cases, and high mortality [6].

More comprehensive coverage of COVID vaccines in the global population is helping to create an immunity barrier against the rise of a new wave. However, an increase in the immune escape potential of emerging variants causes a grave concern for vaccine breakthroughs and reinfections [3,4,7]. With the waning of immunity derived from vaccines and previous infections [8], the risk of the emergence of a more lethal variant capable of creating a global wave remains high and therefore demands continued surveillance [9].

The Delta variant–driven wave showed a rapid peak and fall to the baseline, making it ideal for prediction studies. The Delta strain was first reported from India [10]. Of note, India witnessed a devastating second COVID wave that began toward the end of February 2021 [11]. The unexpected arrival of the second COVID wave, accompanied by an exponential increase in infections, brought the country’s epidemic response system and health infrastructure to a standstill [11], and resulted in massive suffering and loss of life [12].

The Delta variant belongs to the SARS-CoV-2 lineage B.1.617, which appeared as a precursor. The first case of the B.1.617 variant was also reported from India as early as October 2020 [13]. The World Health Organization (WHO) recognized the B.1.617 lineage as a global variant of concern (VOC). The strain evolved into 3 more sublines, namely, B.1.617.1-3, of which B.1.617.1 (the Kappa variant) was declared a variant of interest (VOI) and B.1.617.2 was later declared a VOC by the WHO [14]. B.1.617 contained mutations in key spike protein regions involved in host interactions and the induction of neutralizing antibodies (S: L452R, E484Q, D614G, del681, and del1072) [15]. The sublineages contained lineage-defining spike mutations (L452R and D614G) as well as newly developed mutations as follows: B.1.617.1 (S: T95I, G142D, E154K, L452R, E484Q, D614G, P681R, and Q1071H); B.1.617.2 (S: T19R, G142D, 156del, 157del, R158G, L452R, T478K, D614G, P681R, D614G, P681R, and D950N); and B.1.617.3 (S: T19R, L452R, E484Q, D614G, and P681R) [16]. Contemporary studies suggested that B.1.617 lineage variants were more easily transmissible [13,17-21] and deadlier [18] than the B.1.1.7 lineage (Alpha variant), a globally dominant strain before the second wave [10]. Studies also showed a significant reduction in the neutralization of variants of the B.1.617 lineage by antibodies derived from natural infections and many currently used COVID-19 vaccines, and multiple monoclonal antibodies [18-21]. Notably, B.1.617.2 showed very high transmissibility and immunological escape [10,13,17,22].

Several studies worldwide have shown that predicting an impending COVID-19 wave is possible [23-28]. These studies used mathematical modeling of epidemiological data. Unfortunately, none of them could accurately anticipate a COVID-19 wave. The ability to predict an established wave from epidemiological data alone seems severely limited [12,29].

The analysis of SARS-CoV-2 genomic sequences has emerged as an efficient surveillance tool for understanding the emergence of new variants and their spread. Fortunately, millions of SARS-CoV-2 genomic sequences from regions worldwide are being made publicly available as a collaborative effort to contain the pandemic [30]. The easy availability of high-quality viral sequences with patient metadata has opened a new avenue for potential predictions of the COVID-19 pandemic [31]. However, viral genomic sequences alone may not be sufficient for efficient predictions, and their current uses for this purpose are constrained.

In this study, we propose an integrated approach using viral genome surveillance and epidemiological data for the prediction of an impending COVID-19 wave. We retrospectively analyzed viral genomic sequences and epidemiological data reflecting the emergence and spread of the second wave of COVID-19 in India to construct such a model.

## Methods

### Study Design, Participants, and Data Sources

We analyzed the time series (weekly and monthly) distributions of SARS-CoV-2 variants coupled with epidemiological data from December 1, 2020, to July 26, 2021 (34 weeks) for new cases and deaths from COVID-19 in India. Further, a phylodynamic analysis for individual variants was performed.

We downloaded SARS-CoV-2 genomic sequence data and epidemiological data from the EpiCoV database of the Global Initiative on Sharing All Influenza Data (GISAID) [32] and the Worldometer database [33], respectively. A total of 40,359 genomic sequences of SARS-CoV-2 were analyzed. The sequence for each SARS-CoV-2 variant was retrieved using an automated search function that entered lineage and sublineage information into the EpiCoV database. The total numbers of sequences per week and month for the variants and their relative proportions were calculated (in percentage). The data were tabulated, and each variant’s weekly and monthly distributions were compared to COVID-19 epidemiological data (new cases and deaths) and statistically analyzed. The genomic sequences of SARS-CoV-2 variants in each state and union territory were also examined to check deviations from overall patterns in data.

### Phylodynamics of SARS-CoV-2 Variants 

A phylodynamic analysis of the variants circulating in the Indian population during the study period was performed on GISAID sequences using the bioinformatics tool available at EpiCoV. 

### Statistical Analysis

XLSTAT (Addinsoft) was used to perform all statistical analyses. Descriptive statistics were calculated for each variable. Levene and Anderson tests were used to determine the homogeneity or normality of the data. In addition, a correlation matrix was constructed, and a linear regression analysis was performed between contrasting variables (R values = −1 to +1). Finally, the statistical significance level for each comparison was set at *P*<.05.

### Ethical Considerations

Approval from the institutional ethics committee was not required as the data used in this study were retrieved from publicly available databases.

## Results

**Figure 1:**
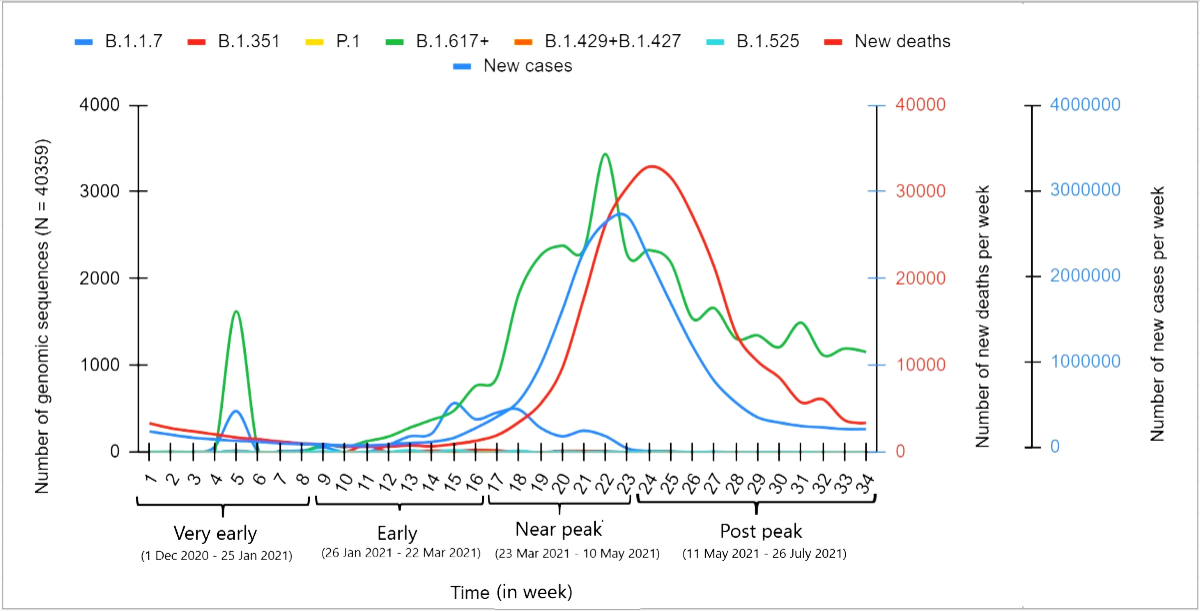
Weekly distribution of SARS-CoV-2 variants in genomic sequence data from India and the correlation with daily new COVID-19 cases and deaths from December 1, 2020, to July 26, 2021. The data were analyzed for the period before the peak of the second wave (23rd week) and after that. SARS-CoV-2 genomic sequence data were obtained from the EpiCoV database of the Global Initiative on Sharing All Influenza Data, and epidemiological data were obtained from the Worldometer database.

Our retrospective analysis of the epidemiological data reflected that the second COVID-19 wave started rising by the end of February 2021 and peaked by the end of the first week of May 2021. Based on the distinct epidemiological trends observed (Multimedia Appendix 1), we divided the study period (December 1, 2020, to July 26, 2021; 34 weeks) into prepeak (weeks 1-23) and postpeak (weeks 24-34) periods. The weekly average of new cases and deaths showed a strong correlation in the study period (R=0.98, *P*<.001), signifying the high statistical validity of the data for further comparisons. Further, we analyzed the distribution of SARS-CoV-2 variants circulating in the Indian population in correlation with new cases and deaths before and after the peak. For description, based on epidemiological trends, the prepeak period was further divided into the following 3 time series intervals: “very early” (weeks 1-8), “early” (weeks 9-16), and “near peak” (weeks 17-23). New cases and deaths showed a downward trend in the “very early” period and maintained a plateau in the “early” period (except toward the end when cases and deaths started increasing, indicating the start of the second wave). In the “near peak” period, a steep rise in new cases and deaths was observed (Figure 1).

**Figure 2:**
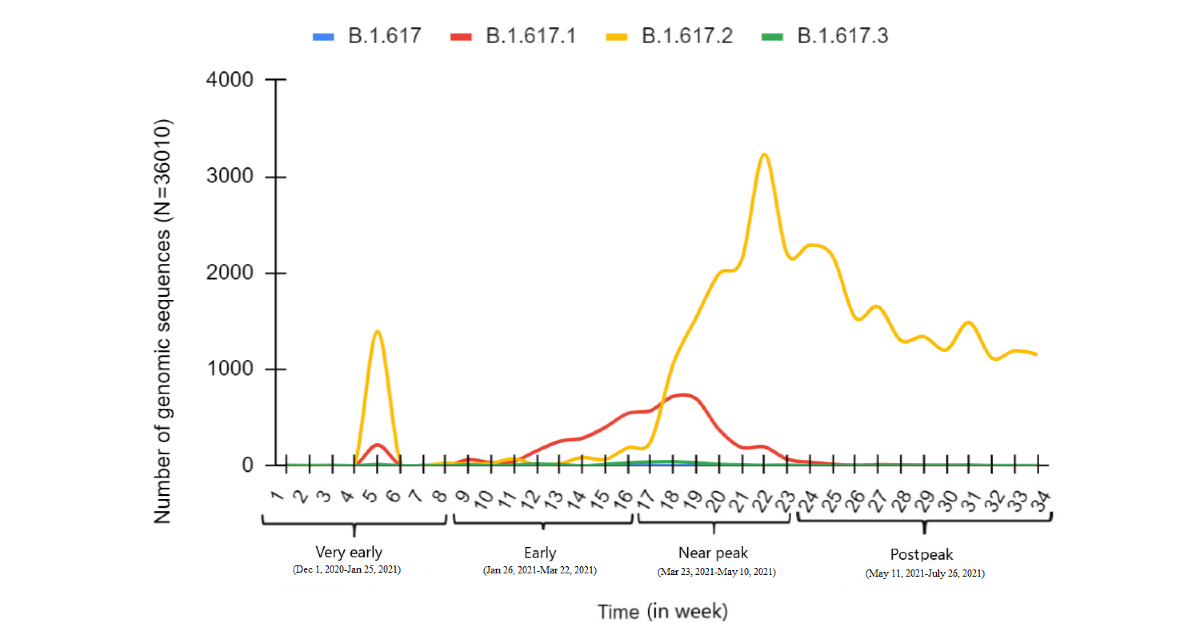
Origin and spread of B.1.617 lineage SARS-CoV-2 variants in the Indian population. Data were analyzed from December 1, 2020, to July 26, 2021. SARS-CoV-2 genomic sequence data were obtained from the EpiCoV database of the Global Initiative on Sharing All Influenza Data, and epidemiological data were obtained from the Worldometer database.

The rise and fall of circulating SARS-CoV-2 variants were studied against the observed epidemiological data trends in the respective time series intervals. Observing the composite data trends of epidemiological and SARS-CoV-2 genomic data provides a glimpse of the formation of the second COVID-19 wave, with clear indications of which SARS-CoV-2 strains may have driven it (Figures 1 and 2). By December 2020, 8 SARS-CoV-2 Pango lineages and their multiple sublineages were circulating in the Indian population, including 4 VOCs (B.1.1.7, B.1.351, P1, and B.1.617.2) and 3 VOIs (B.1.617.1, B.1.127/B.1.429, and B.1.525). However, B.1.1.7 was the most dominant variant in that period. B.1.617 lineage variants collectively (B.1.617+) showed an upward trend since their emergence, and surpassed other VOCs, including B.1.1.7, by the end of January 2021 (weeks 8-9) and subsequently kept rising. In contrast, B.1.1.7 showed a downward trend by the end of March 2021 (weeks 17-18), with B.1.617 lineage variants becoming the dominant variants. By the end of April 2021, B.1.617 lineage variants were detected in 78.5% of SARS-CoV-2 sequences uploaded on the GISAID database, reaching about 83% in the week of the peak.

**Figure 3:**
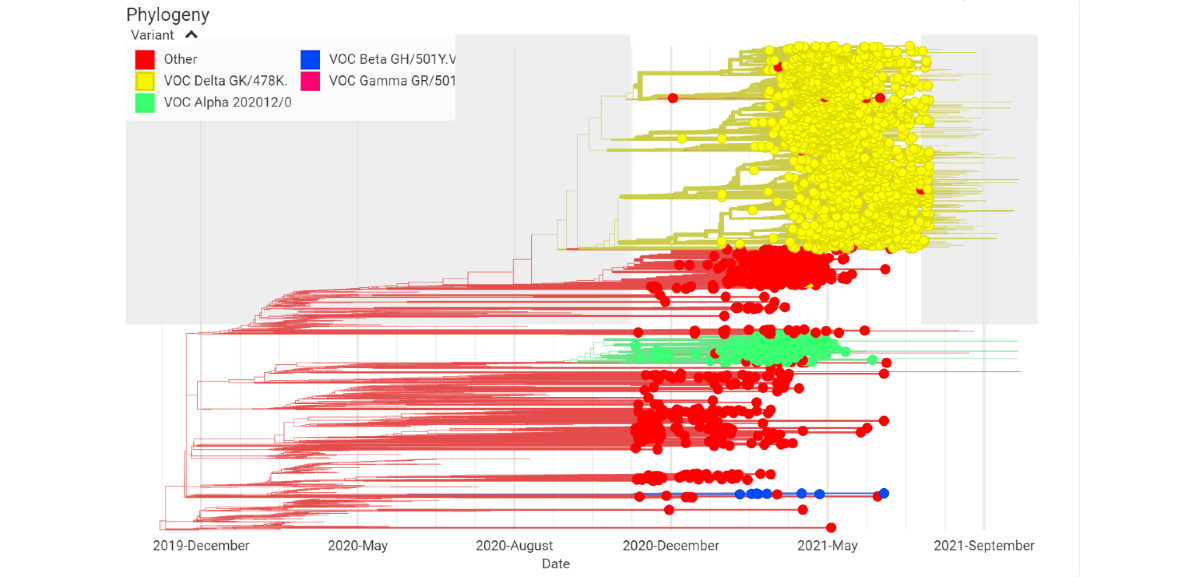
Phylodynamics of SARS-CoV-2 variants in the Indian population from December 1, 2020, to July 26, 2021. SARS-CoV-2 genomic sequence data were obtained from the EpiCoV database of the Global Initiative on Sharing All Influenza Data, and epidemiological data were obtained from the Worldometer database. VOC: variant of concern.

The phylodynamic analysis of the circulating variants in the study period strongly corroborated the trends present in the graph data, showing an exclusive increase in the cluster density of B.1.617.2 compared with other variants in the “near peak” period (Figure 3).

To know whether the rise in the B.1.617.2 variant was localized to specific geographical regions, which may have influenced the collective data trends, we compared the monthly distribution of genomic sequences of SARS-CoV-2 variants for the states and union territories of India individually. A similar increase in the detection of the B.1.617.2 variant was observed in most states and union territories (Multimedia Appendix 2), except Kerala, where different patterns were visible (Figure S15 in Multimedia Appendix 2). In Kerala, the rise of the B.1.617.2 variant was slower in comparison with the rest of the country (55.5% vs 72% of total cases by the end of April 2021), which was further confirmed in the state-wise serosurvey data from the period of the second wave (44.4% vs 67.7% of the national average) [34]. Notably, a sharp rise in B.1.617.2 cases was observed in Kerala in a later period.

## Discussion

### Principal Findings

The retrospective examination of linked viral genomic sequences and epidemiological data in this study clearly showed that the occurrence of B.1.617 lineage variants, particularly the B.1.617.2 sublineage, was strongly related to the second wave of COVID-19 in India. In late January 2021, when instances of B.1.617.2 surpassed those of all other variants, the first signs of an imminent wave of COVID-19 began to appear. The rise of the wave could be observed closely until the end of March 2021, when instances of B.1.617.2 showed a sharp increase in line with the total number of new cases.

### Comparison With Prior Work

Current prediction models in the COVID-19 pandemic are dominated by purely epidemiological analyses, from which hardly anyone could accurately predict an impending COVID-19 wave [23-27]. The importance of studying viral genomic sequences for the epidemiological surveillance of new SARS-CoV-2 variants is well recognized [31,35-40]. However, its application in developing a predictive model to forecast upcoming virus waves has received little appreciation in the existing literature [41]. Interestingly, strong conceptual validation for the applicability of an integrated approach to predict an impending COVID-19 wave using viral genomic surveillance and epidemiological data came from a recent study by de Hoffer et al [42]. These authors studied the temporal dynamics of emerging SARS-CoV-2 variants using a machine learning algorithm–based analysis of the spike protein sequences of viral samples from England, Scotland, and Wales reported in the GISAID database. Further, they correlated the relative percentage of each variant with the weekly and monthly epidemiological data of active cases from the studied geographical regions. They showed a strong relationship between the genesis of a new emerging variant and the onset of a new wave, with an exponential increase in the number of infections [42]. 

Moreover, our findings regarding the second wave of COVID-19 in India are corroborated by a previous study by Dhar et al [10]. The authors analyzed viral genomic sequences retrospectively and observed a similar pattern in the rise of the B.1.617 lineage, mainly the B.1.617.2 variant, in Delhi before the second wave [10]. A B.1.617.2-driven second wave was also reflected in the analysis of viral genomic sequences performed by Adiga and Nayak in 2021 [43]. We recently used our prediction model prospectively during the initial rise of cases caused by the Omicron strain in South Africa, which indicated an upcoming wave with very high transmissibility but limited lethality [4]. These predictions were later accurately reflected in the studies reporting the Omicron-mediated fourth wave of COVID-19 in South Africa [44,45].

The potential predictability of the second wave of COVID-19 in India in the retrospective data analysis suggests that genomic surveillance of SARS-CoV-2 variants, enriched with epidemiological data, could be a potential tool to predict upcoming COVID-19 waves. Still, the prediction accuracy is largely dependent on population-based viral genomic sequencing and consistency in data upload from all geographic regions, as well as accurate reporting of epidemiological data. The sole increase in the proportion of an emerging SARS-CoV-2 variant, coupled with an associated rise in new cases, might inform the arrival of a new wave of COVID-19. However, consideration of other epidemiological factors, such as previous exposure to related virus strains and the immunization status of the population, will be necessary to determine the magnitude of an impending wave [46]. Notably, the first wave of COVID-19 in India was limited in scope, as evidenced by the serosurvey data [47,48], and only a small part of the population was vaccinated as of early 2021 [49]. With the emergence of a new variant, both these factors may have created an ideal environment for a massive second wave to emerge. In addition, preventive measures, such as blocking or limiting gatherings and using face masks, can also influence the prospects and magnitude of a new wave [29].

### Limitations

There were some limitations in our study that may have influenced the interpretation of the results. First, the samples used in our analyses might not be representative of the population. In many geographical regions, the sample size was grossly disproportionate. Therefore, the genomic sequence data presented in this study might not reflect the exact epidemiological extent of the distribution of the variants in the reported geographical regions but only show their relative proportions in the samples for which genomic sequences were uploaded to the GISAID database. We have assumed that similar proportions exist between variants in the actual population. Second, inconsistent reports and uploads of genomic sequences made it challenging to study a daily trend in the spread of variants. Finally, the scarcity of genomic sequences and inconsistency in uploading to the databases used for some states/union territories made determining variant dominance difficult.

### Conclusions

Based on the observations of this study, we propose that genomic surveillance of SARS-CoV-2 variants, complemented with epidemiological data, can be a promising tool to predict upcoming COVID-19 waves.

## Appendix

Multimedia Appendix 1 Weekly new COVID-19 cases and deaths in the Indian population for the period of December 1, 2020, to July 26, 2021.

Multimedia Appendix 2 Monthly distribution of SARS-CoV-2 variants in genomic sequence data from the states and union territories of India uploaded on the EpiCoV database for the period of December 1, 2020, to July 26, 2021.

## Abbreviations

GISAID: Global Initiative on Sharing All Influenza Data
VOC: variant of concern
VOI: variant of interest
WHO: World Health Organization

## Data Availability

The primary data used for this study are publicly available on the EpiCoV database of the Global Initiative on Sharing All Influenza Data (SARS-CoV-2 genomic sequence data) and the Worldometer database (epidemiological data). The categorized data for the study period can be availed from the corresponding author on reasonable request.

## References

[1] Coronavirus Statistics. Worldometers.

[2] O'Brien DA, Clements C (2021). Early warning signal reliability varies with COVID-19 waves. Biol Lett.

[3] Kumar A, Parashar R, Kumar S, Faiq MA, Kumari C, Kulandhasamy M, Narayan RK, Jha RK, Singh HN, Prasoon P, Pandey SN, Kant K (2022). Emerging SARS-CoV-2 variants can potentially break set epidemiological barriers in COVID-19. J Med Virol.

[4] Kumar A, Asghar A, Singh H, Faiq M, Kumar S, Narayan R, Kumar G, Dwivedi P, Sahni C (2021). An in silico analysis of early SARS-CoV-2 variant B.1.1.529 (Omicron) genomic sequences and their epidemiological correlates. medRxiv.

[5] Tareq A, Emran TB, Dhama K, Dhawan M, Tallei T (2021). Impact of SARS-CoV-2 delta variant (B.1.617.2) in surging second wave of COVID-19 and efficacy of vaccines in tackling the ongoing pandemic. Hum Vaccin Immunother.

[6] Ong S, Chiew C, Ang L, Mak T, Cui L, Toh M, Lim YD, Lee PH, Lee TH, Chia PY, Maurer-Stroh S, Lin RTP, Leo YS, Lee VJ, Lye DC, Young BE (2022). Clinical and Virological Features of Severe Acute Respiratory Syndrome Coronavirus 2 (SARS-CoV-2) Variants of Concern: A Retrospective Cohort Study Comparing B.1.1.7 (Alpha), B.1.351 (Beta), and B.1.617.2 (Delta). Clin Infect Dis.

[7] Guo Y, Han J, Zhang Y, He J, Yu W, Zhang X, Wu J, Zhang S, Kong Y, Guo Y, Lin Y, Zhang J (2022). SARS-CoV-2 Omicron Variant: Epidemiological Features, Biological Characteristics, and Clinical Significance. Front Immunol.

[8] Ferdinands JM, Rao S, Dixon BE, Mitchell PK, DeSilva MB, Irving SA, Lewis N, Natarajan K, Stenehjem E, Grannis SJ, Han J, McEvoy C, Ong TC, Naleway AL, Reese SE, Embi PJ, Dascomb K, Klein NP, Griggs EP, Konatham D, Kharbanda AB, Yang DH, Fadel WF, Grisel N, Goddard K, Patel P, Liao IC, Birch R, Valvi NR, Reynolds S, Arndorfer J, Zerbo O, Dickerson M, Murthy K, Williams J, Bozio CH, Blanton L, Verani JR, Schrag SJ, Dalton AF, Wondimu MH, Link-Gelles R, Azziz-Baumgartner E, Barron MA, Gaglani M, Thompson MG, Fireman B (2022). Waning 2-Dose and 3-Dose Effectiveness of mRNA Vaccines Against COVID-19-Associated Emergency Department and Urgent Care Encounters and Hospitalizations Among Adults During Periods of Delta and Omicron Variant Predominance - VISION Network, 10 States, August 2021-January 2022. MMWR Morb Mortal Wkly Rep.

[9] (2022). Rapidly escalating COVID-19 cases amid reduced virus surveillance forecasts a challenging autumn and winter in the WHO European Region. World Health Organization.

[10] Dhar MS, Marwal R, Vs R, Ponnusamy K, Jolly B, Bhoyar RC, Sardana V, Naushin S, Rophina M, Mellan TA, Mishra S, Whittaker C, Fatihi S, Datta M, Singh P, Sharma U, Ujjainiya R, Bhatheja N, Divakar MK, Singh MK, Imran M, Senthivel V, Maurya R, Jha N, Mehta P, Sharma P, Vr A, Chaudhary U, Soni N, Thukral L, Flaxman S, Bhatt S, Pandey R, Dash D, Faruq M, Lall H, Gogia H, Madan P, Kulkarni S, Chauhan H, Sengupta S, Kabra S, Gupta RK, Singh SK, Agrawal A, Rakshit P, Nandicoori V, Tallapaka KB, Sowpati DT, Thangaraj K, Bashyam MD, Dalal A, Sivasubbu S, Scaria V, Parida A, Raghav SK, Prasad P, Sarin A, Mayor S, Ramakrishnan U, Palakodeti D, Seshasayee ASN, Bhat M, Shouche Y, Pillai A, Dikid T, Das S, Maitra A, Chinnaswamy S, Biswas NK, Desai AS, Pattabiraman C, Manjunatha MV, Mani RS, Arunachal Udupi G, Abraham P, Atul PV, Cherian SS, Indian SARS-CoV-2 Genomics Consortium (INSACOG) (2021). Genomic characterization and epidemiology of an emerging SARS-CoV-2 variant in Delhi, India. Science.

[11] Samarasekera U (2021). India grapples with second wave of COVID-19. The Lancet Microbe.

[12] Jha P, Deshmukh Y, Tumbe C, Suraweera W, Bhowmick A, Sharma S, Novosad P, Fu SH, Newcombe L, Gelband H, Brown P (2022). COVID mortality in India: National survey data and health facility deaths. Science.

[13] Planas D, Veyer D, Baidaliuk A, Staropoli I, Guivel-Benhassine F, Rajah M, Planchais C, Porrot F, Robillard N, Puech J, Prot M, Gallais F, Gantner P, Velay A, Le Guen J, Kassis-Chikhani N, Edriss D, Belec L, Seve A, Courtellemont L, Péré H, Hocqueloux L, Fafi-Kremer S, Prazuck T, Mouquet H, Bruel T, Simon-Lorière E, Rey FA, Schwartz O (2021). Reduced sensitivity of SARS-CoV-2 variant Delta to antibody neutralization. Nature.

[14] Tracking SARS-CoV-2 variants. World Health Organization.

[15] B.1.617.2) Lineage Report. Outbreak.info.

[16] Weekly epidemiological update on COVID-19 - 18 May 2021. World Health Organization.

[17] Lopez Bernal J, Andrews N, Gower C, Gallagher E, Simmons R, Thelwall S, Stowe J, Tessier E, Groves N, Dabrera G, Myers R, Campbell CN, Amirthalingam G, Edmunds M, Zambon M, Brown KE, Hopkins S, Chand M, Ramsay M (2021). Effectiveness of Covid-19 Vaccines against the B.1.617.2 (Delta) Variant. N Engl J Med.

[18] Yadav P, Mohandas S, Shete A, Nyayanit D, Gupta N, Patil D, Sapkal GN, Potdar V, Kadam M, Kumar A (2021). SARS CoV-2 variant B.1.617.1 is highly pathogenic in hamsters than B.1 variant. bioRxiv.

[19] Tada T, Zhou H, Dcosta BM, Samanovic MI, Mulligan MJ, Landau NR (2021). Partial resistance of SARS-CoV-2 Delta variants to vaccine-elicited antibodies and convalescent sera. iScience.

[20] Hoffmann M, Hofmann-Winkler H, Krüger N, Kempf A, Nehlmeier I, Graichen L, Arora P, Sidarovich A, Moldenhauer AS, Winkler MS, Schulz S, Jäck HM, Stankov MV, Behrens GMN, Pöhlmann S (2021). SARS-CoV-2 variant B.1.617 is resistant to bamlanivimab and evades antibodies induced by infection and vaccination. Cell Rep.

[21] Ferreira I, Datir R, Papa G, Kemp S, Meng B, Rakshit P, Singh S, Pandey R, Ponnusamy K (2021). SARS-CoV-2 B.1.617 emergence and sensitivity to vaccine-elicited antibodies. bioRxiv.

[22] Kumar A, Asghar A, Raza K, Narayan R, Jha R, Satyam A, Kumar G, Dwivedi P, Sahni C (2021). Demographic characteristics of SARS-CoV-2 B.1.617.2 (Delta) variant infections in Indian population. medRxiv.

[23] Kolozsvári LR, Bérczes T, Hajdu A, Gesztelyi R, Tiba A, Varga I, Al-Tammemi AB, Szőllősi GJ, Harsányi S, Garbóczy S, Zsuga J (2021). Predicting the epidemic curve of the coronavirus (SARS-CoV-2) disease (COVID-19) using artificial intelligence: An application on the first and second waves. Inform Med Unlocked.

[24] Kibria H, Jyoti O, Matin A (2022). Forecasting the spread of the third wave of COVID-19 pandemic using time series analysis in Bangladesh. Inform Med Unlocked.

[25] Sharif O, Hasan M, Rahman A (2022). Determining an effective short term COVID-19 prediction model in ASEAN countries. Sci Rep.

[26] Mohan S, Solanki A, Taluja H, Singh A, Anuradha (2022). Predicting the impact of the third wave of COVID-19 in India using hybrid statistical machine learning models: A time series forecasting and sentiment analysis approach. Comput Biol Med.

[27] Yang Z, Zeng Z, Wang K, Wong S, Liang W, Zanin M, Liu P, Cao X, Gao Z, Mai Z, Liang J, Liu X, Li S, Li Y, Ye F, Guan W, Yang Y, Li F, Luo S, Xie Y, Liu B, Wang Z, Zhang S, Wang Y, Zhong N, He J (2020). Modified SEIR and AI prediction of the epidemics trend of COVID-19 in China under public health interventions. J Thorac Dis.

[28] Thakur S, Patel D, Soni B, Raval M, Chaudhary S, Bellatreche L, Goyal V, Fujita H, Mondal A, Reddy PK (2020). Prediction for the Second Wave of COVID-19 in India. Big Data Analytics. BDA 2020. Lecture Notes in Computer Science, vol 12581.

[29] Salvatore M, Purkayastha S, Ganapathi L, Bhattacharyya R, Kundu R, Zimmermann L, Ray D, Hazra A, Kleinsasser M, Solomon S, Subbaraman R, Mukherjee B (2022). Lessons from SARS-CoV-2 in India: A data-driven framework for pandemic resilience. Sci Adv.

[30] Chen Z, Azman AS, Chen X, Zou J, Tian Y, Sun R, Xu X, Wu Y, Lu W, Ge S, Zhao Z, Yang J, Leung DT, Domman DB, Yu H (2022). Global landscape of SARS-CoV-2 genomic surveillance and data sharing. Nat Genet.

[31] Oude Munnink BB, Worp N, Nieuwenhuijse D, Sikkema R, Haagmans B, Fouchier R, Koopmans M (2021). The next phase of SARS-CoV-2 surveillance: real-time molecular epidemiology. Nat Med.

[32] Global Initiative on Sharing All Influenza Data (GISAID).

[33] India coronavirus data. Worldometer.

[34] Dasgupta R After India’s brutal coronavirus wave, two-thirds of population has been exposed to SARS-CoV2. The Conversation.

[35] Smith M, Trofimova M, Weber A, Duport Y, Kühnert D, von Kleist M (2021). Rapid incidence estimation from SARS-CoV-2 genomes reveals decreased case detection in Europe during summer 2020. Nat Commun.

[36] Yadav PD, Nyayanit DA, Majumdar T, Patil S, Kaur H, Gupta N, Shete AM, Pandit P, Kumar A, Aggarwal N, Narayan J, Vijay N, Kalawat U, Sugunan AP, Munivenkatappa A, Sharma T, Devi S, Majumdar T, Jaryal S, Bakshi R, Joshi Y, Sahay R, Shastri J, Singh M, Kumar M, Rawat V, Dutta S, Yadav S, Krishnasamy K, Raut S, Biswas D, Borkakoty B, Verma S, Rani S, Deval H, Patel D, Turuk J, Malhotra B, Fomda B, Nag V, Jain A, Bhargava A, Potdar V, Cherian S, Abraham P, Gopal A, Panda S, Bhargava B (2021). An Epidemiological Analysis of SARS-CoV-2 Genomic Sequences from Different Regions of India. Viruses.

[37] Long S, Olsen R, Christensen P, Bernard D, Davis J, Shukla M, Nguyen M, Saavedra MO, Yerramilli P, Pruitt L, Subedi S, Kuo HC, Hendrickson H, Eskandari G, Nguyen HAT, Long JH, Kumaraswami M, Goike J, Boutz D, Gollihar J, McLellan JS, Chou CW, Javanmardi K, Finkelstein IJ, Musser JM (2020). Molecular Architecture of Early Dissemination and Massive Second Wave of the SARS-CoV-2 Virus in a Major Metropolitan Area. mBio.

[38] Ahammad I, Hossain M, Rahman A, Chowdhury Z, Bhattacharjee A, Das K, Keya CA, Salimullah M (2021). Wave-wise comparative genomic study for revealing the complete scenario and dynamic nature of COVID-19 pandemic in Bangladesh. PLoS One.

[39] Maher M, Bartha I, Weaver S, di Iulio J, Ferri E, Soriaga L, Lempp FA, Hie BL, Bryson B, Berger B, Robertson DL, Snell G, Corti D, Virgin HW, Kosakovsky Pond SL, Telenti A (2022). Predicting the mutational drivers of future SARS-CoV-2 variants of concern. Sci Transl Med.

[40] Nagpal S, Pal R, Tyagi A, Tripathi S, Nagori A, Ahmad S, Mishra HP, Malhotra R, Kutum R, Sethi T, Ashima (2022). Genomic Surveillance of COVID-19 Variants With Language Models and Machine Learning. Front Genet.

[41] Hill V, Ruis C, Bajaj S, Pybus O, Kraemer M (2021). Progress and challenges in virus genomic epidemiology. Trends Parasitol.

[42] de Hoffer A, Vatani S, Cot C, Cacciapaglia G, Chiusano M, Cimarelli A, Conventi F, Giannini A, Hohenegger S, Sannino F (2022). Variant-driven early warning via unsupervised machine learning analysis of spike protein mutations for COVID-19. Sci Rep.

[43] Adiga R, Nayak V (2021). Emergence of Novel SARS-CoV-2 variants in India: second wave. J Infect Dev Ctries.

[44] Maslo C, Friedland R, Toubkin M, Laubscher A, Akaloo T, Kama B (2022). Characteristics and Outcomes of Hospitalized Patients in South Africa During the COVID-19 Omicron Wave Compared With Previous Waves. JAMA.

[45] Jassat W, Abdool Karim SS, Mudara C, Welch R, Ozougwu L, Groome MJ, Govender N, von Gottberg A, Wolter N, Wolmarans M, Rousseau P, Blumberg L, Cohen C (2022). Clinical severity of COVID-19 in patients admitted to hospital during the omicron wave in South Africa: a retrospective observational study. The Lancet Global Health.

[46] Dyson L, Hill E, Moore S, Curran-Sebastian J, Tildesley M, Lythgoe K, House T, Pellis L, Keeling MJ (2021). Possible future waves of SARS-CoV-2 infection generated by variants of concern with a range of characteristics. Nat Commun.

[47] Murhekar MV, Bhatnagar T, Thangaraj JWV, Saravanakumar V, Kumar MS, Selvaraju S, Rade K, Kumar CPG, Sabarinathan R, Turuk A, Asthana S, Balachandar R, Bangar S, Bansal A, Chopra V, Das D, Deb A, Devi K, Dhikav V, Dwivedi G, Khan S, Kumar M, Laxmaiah A, Madhukar M, Mahapatra A, Rangaraju C, Turuk J, Yadav R, Andhalkar R, Arunraj K, Bharadwaj D, Bharti P, Bhattacharya D, Bhat J, Chahal A, Chakraborty D, Chaudhury A, Deval H, Dhatrak S, Dayal R, Elantamilan D, Giridharan P, Haq I, Hudda R, Jagjeevan B, Kalliath A, Kanungo S, Krishnan N, Kshatri J, Kumar A, Kumar N, Kumar V, Lakshmi G, Mehta G, Mishra N, Mitra A, Nagbhushanam K, Nimmathota A, Nirmala A, Pandey A, Prasad G, Qurieshi M, Reddy S, Robinson A, Sahay S, Saxena R, Sekar K, Shukla V, Singh H, Singh P, Singh P, Singh R, Srinivasan N, Varma D, Viramgami A, Wilson V, Yadav S, Yadav S, Zaman K, Chakrabarti A, Das A, Dhaliwal R, Dutta S, Kant R, Khan A, Narain K, Narasimhaiah S, Padmapriyadarshini C, Pandey K, Pati S, Patil S, Rajkumar H, Ramarao T, Sharma Y, Singh S, Panda S, Reddy D, Bhargava B, ICMR Serosurveillance Group (2021). SARS-CoV-2 seroprevalence among the general population and healthcare workers in India, December 2020-January 2021. Int J Infect Dis.

[48] Jahan N, Brahma A, Kumar M, Bagepally B, Ponnaiah M, Bhatnagar T, Murhekar MV (2022). Seroprevalence of IgG antibodies against SARS-CoV-2 in India, March 2020 to August 2021: a systematic review and meta-analysis. Int J Infect Dis.

[49] Chakraborty C, Sharma A, Bhattacharya M, Agoramoorthy G, Lee S (2021). The current second wave and COVID-19 vaccination status in India. Brain Behav Immun.

