# Supplementary material for: A Bioinformatics Tool for Predicting Future COVID-19 Waves Based on a Retrospective Analysis of the Second Wave in India: Model Development Study
Source: JMIR Bioinform Biotechnol. 2022 Sep 22;3(1):e36860. doi: 10.2196/36860 (PMC9516867; doi:10.2196/36860)
Supplement: Multimedia Appendix 1 [file bioinform_v3i1e36860_app1.docx]

**Multimedia Appendix 1.** Weekly new COVID-19 cases and deaths in the Indian population for the period of December 1, 2020, to July 26, 2021.

**
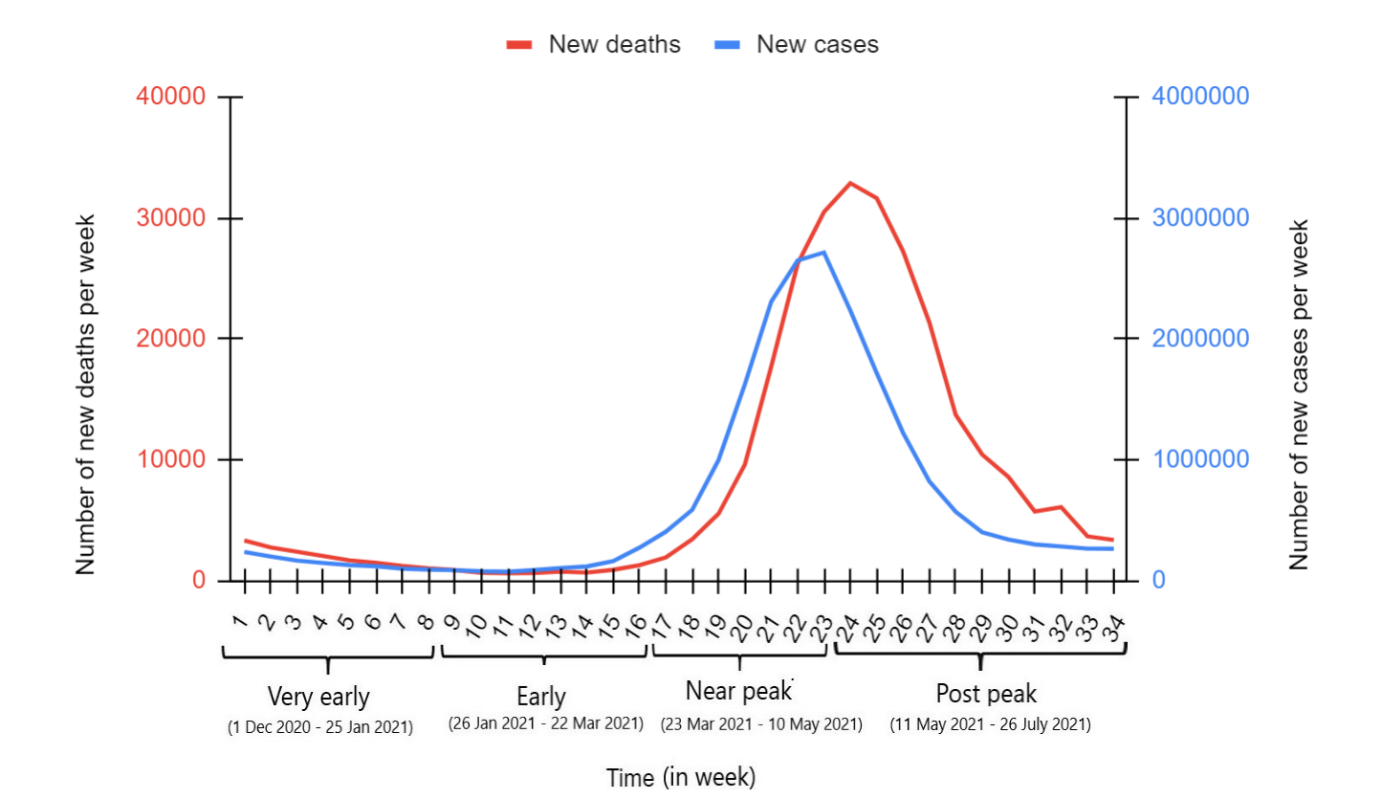
**

The data were analyzed for the period before the peak of the second wave (23rd week) and thereafter. For the purpose of description, based on the epidemiological trends, the pre peak period was further divided into three time series intervals: ‘very early’ (1-8 weeks), ‘early’ (9-16 weeks), and ‘near peak’ (17-23 weeks). (Data source: Worldometer: https://www.worldometers.info/coronavirus/coronavirus/country/india).
