# Supplementary material for: A Bioinformatics Tool for Predicting Future COVID-19 Waves Based on a Retrospective Analysis of the Second Wave in India: Model Development Study
Source: JMIR Bioinform Biotechnol. 2022 Sep 22;3(1):e36860. doi: 10.2196/36860 (PMC9516867; doi:10.2196/36860)
Supplement: Multimedia Appendix 2 [file bioinform_v3i1e36860_app2.docx]

**Multimedia Appendix 2.** Monthly distribution of SARS-CoV-2 variants in genomic sequence data from the states and union territories of India uploaded on the EpiCoV database for the period of December 1, 2020, to July 26, 2021.


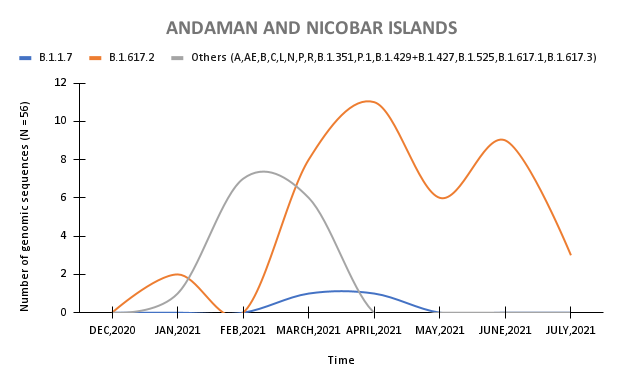


Figure S1


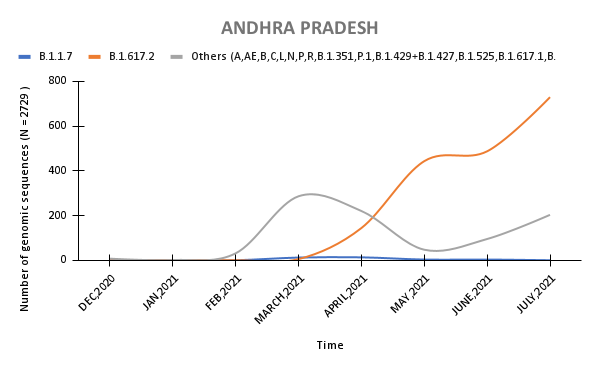


Figure S2


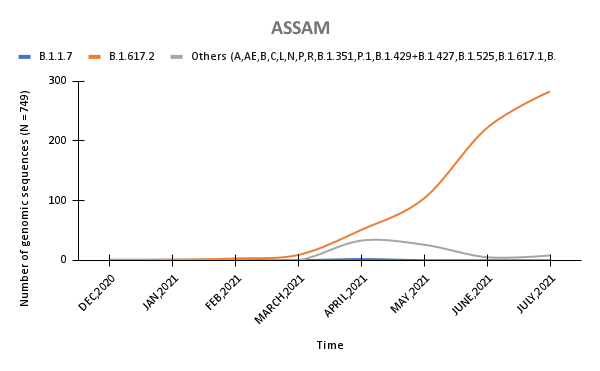


Figure S3


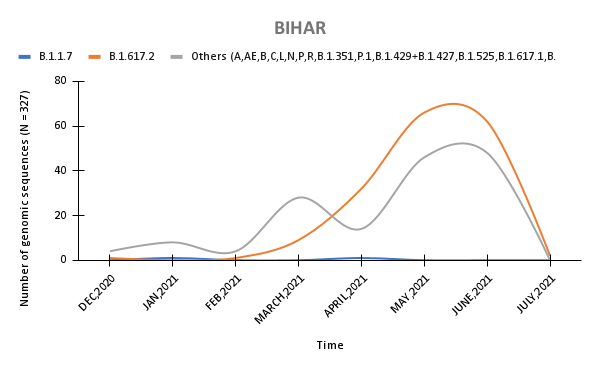


Figure S4


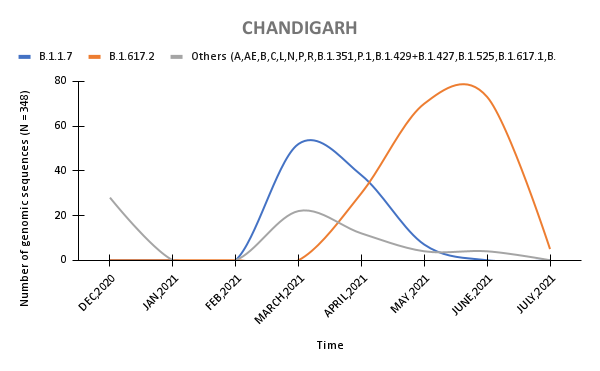


Figure S5


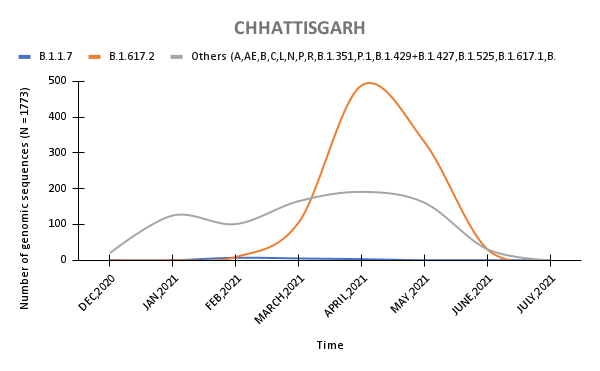


Figure S6


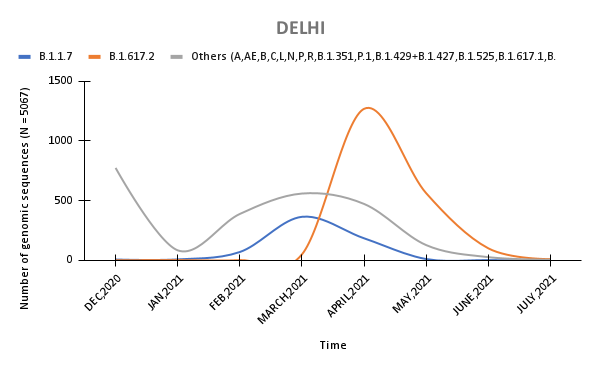


Figure S7


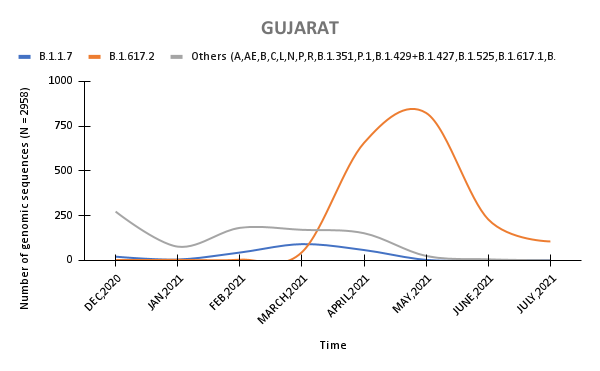


Figure S8


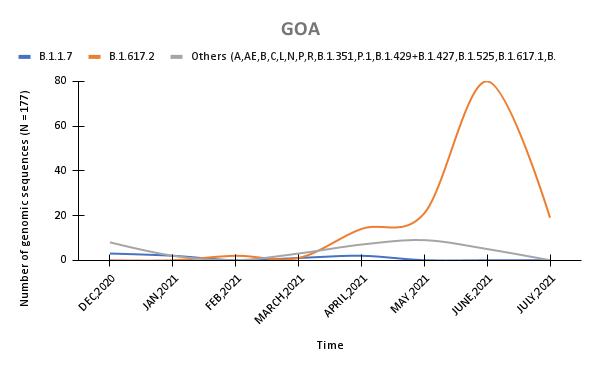


Figure S9


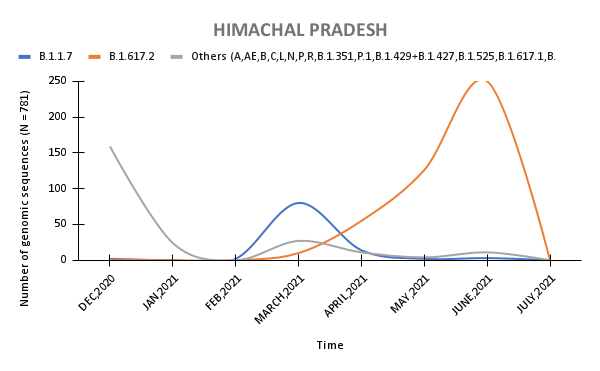


Figure S10


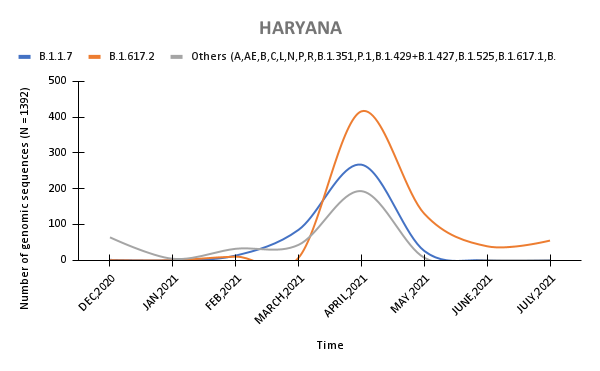


Figure S11


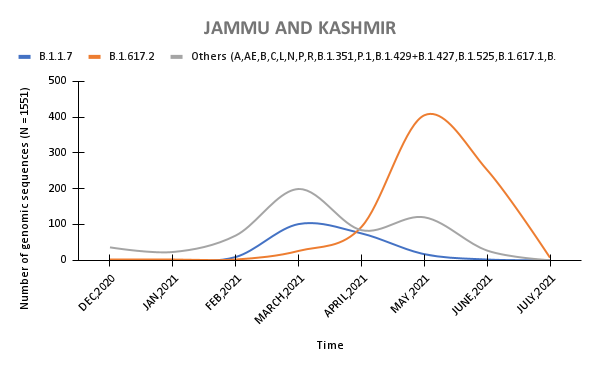


Figure S12


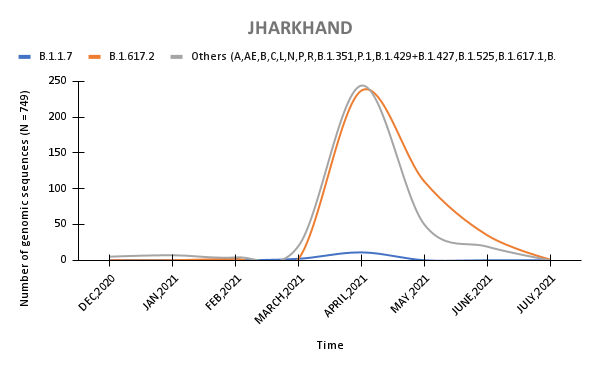


Figure S13


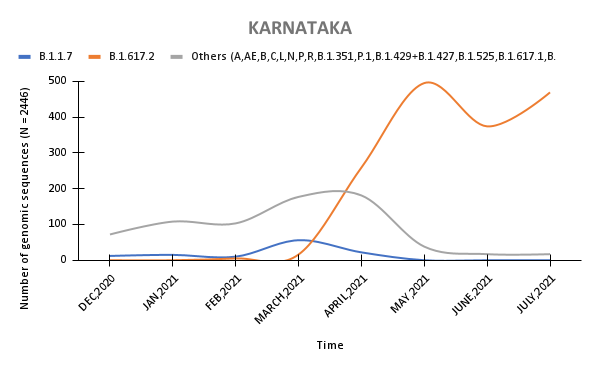


Figure S14


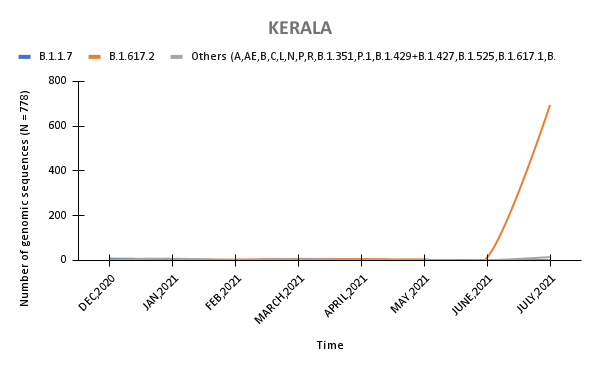


Figure S15


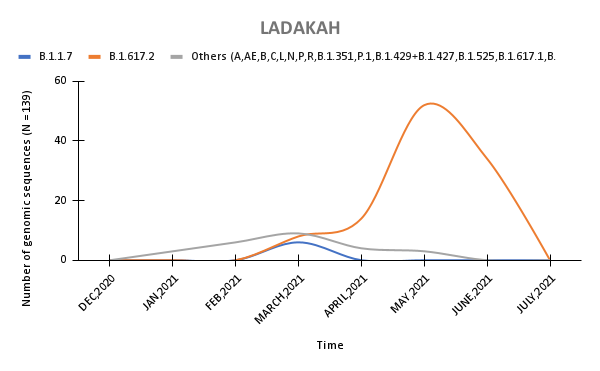


Figure S16


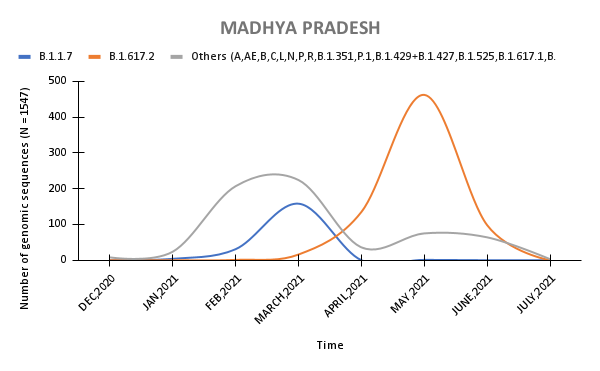


Figure S17


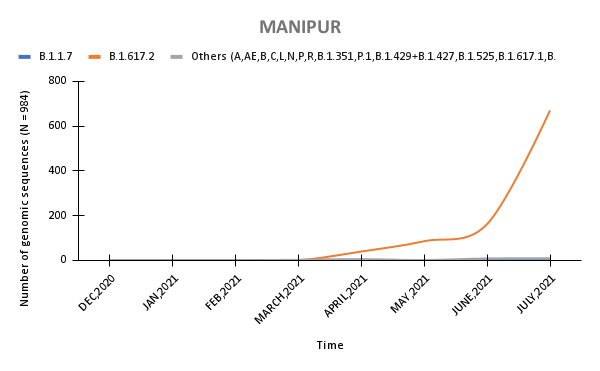


Figure S18


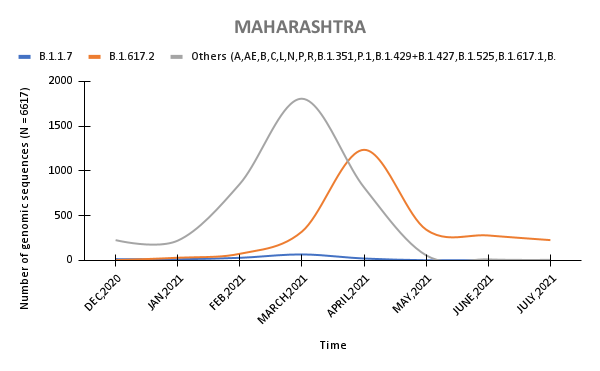


Figure S19


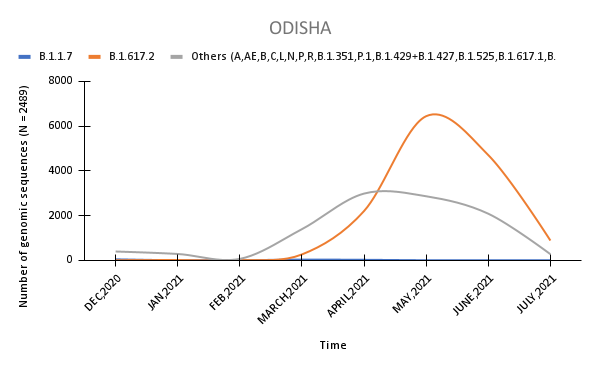


Figure S20


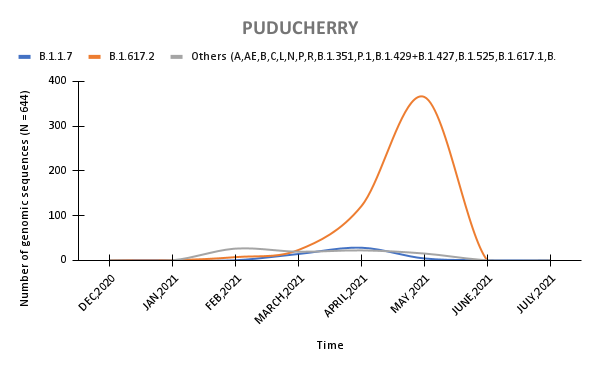


Figure S21


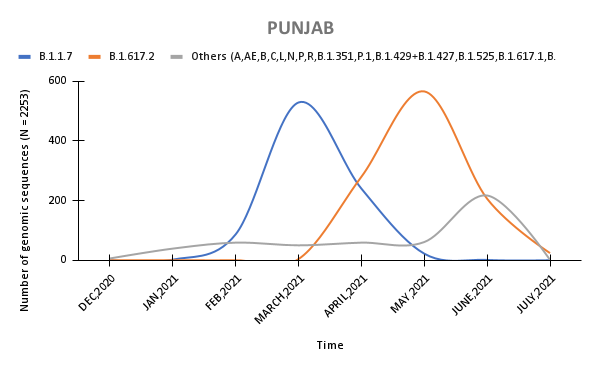


Figure S22


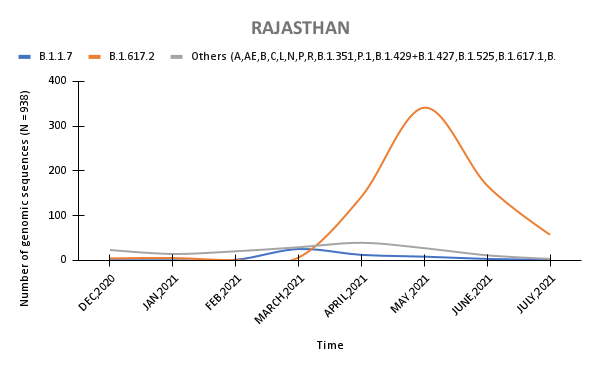


Figure S23


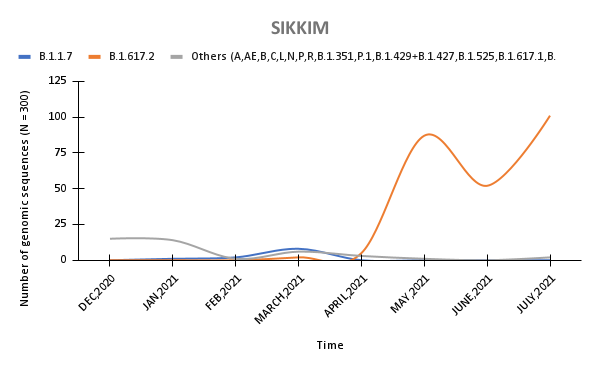


Figure S24


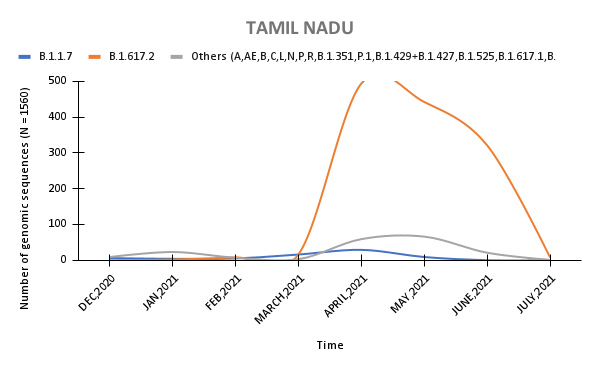


Figure S25


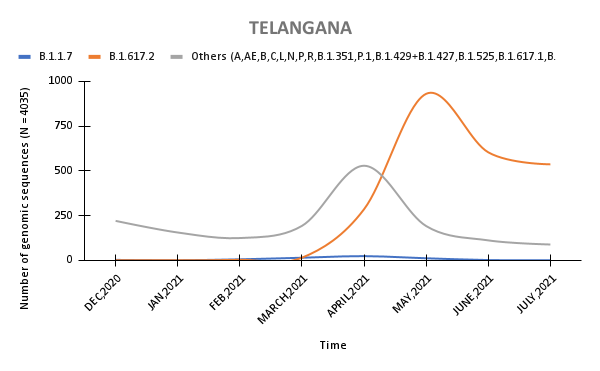


Figure S26


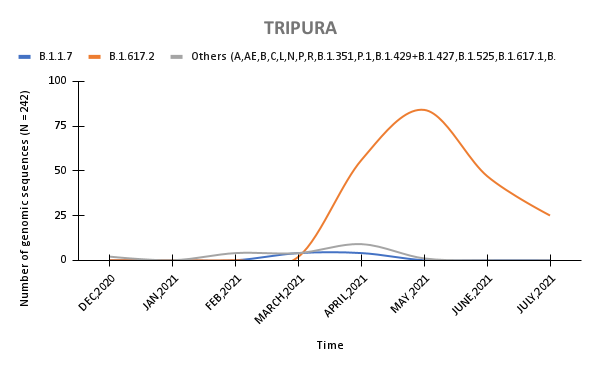


Figure S27


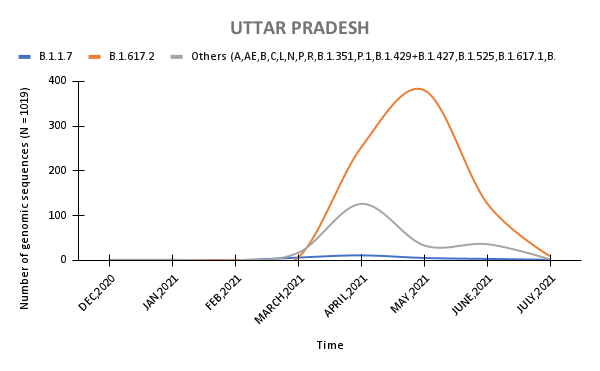


Figure S28


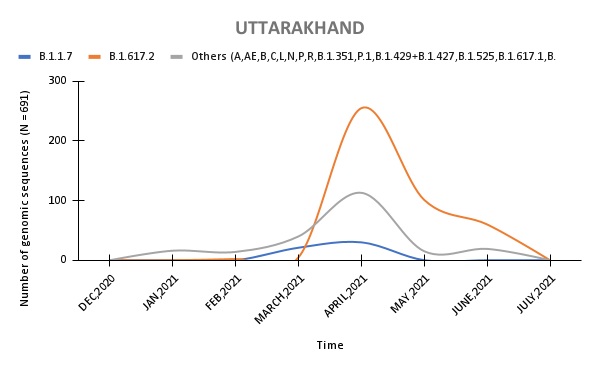


Figure S29


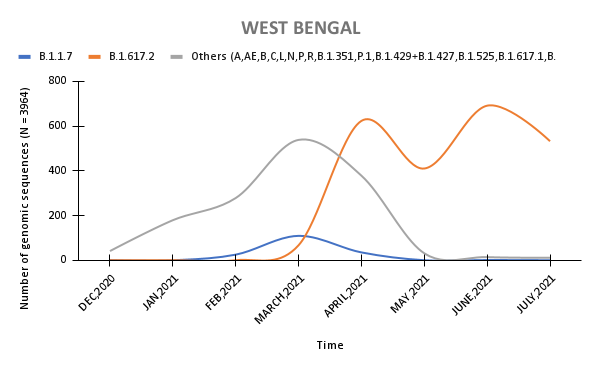


Figure S30
